# The Role of Epigenetics in Myocardial Infarction: Mechanism, Biomarkers and Therapeutic Potential

**DOI:** 10.31661/gmj.v13i.3474

**Published:** 2024-09-11

**Authors:** Arash Amin, Mohsen Abbasnezhad, Ali Keshavarzian, Ahmadreza Badali, Roohollah Rahbani, Reza Faramarz Zadeh

**Affiliations:** ^1^ Lorestan Heart Center (Madani Hospital), Lorestan University of Medical Sciences, Khorram-Abad, Lorestan, Iran; ^2^ Cardiovascular Research Center, Tabriz University of Medical Sciences, Tabriz, Iran; ^3^ Golestan University of Medical Science, Gorgan, Iran; ^4^ I.M. Sechenov First Moscow State Medical University, Moscow, Russia; ^5^ Seyed-Al-Shohada Cardiology Hospital, Urmia University of Medical Sciences, Urmia, Iran

**Keywords:** Myocardial Infarction, Epigenetics, DNA Methylation, Noncoding RNAs, Cardiac Repair

## Abstract

Myocardial infarction (MI), remains one of the leading causes of morbidity and mortality worldwide. As a result, understanding the underlying mechanisms of MI is crucial for developing effective therapeutic strategies. Epigenetics, which involves heritable changes in gene expression without altering the underlying DNA sequence, has emerged as a significant factor in the pathogenesis and progression of MI. Key epigenetic mechanisms such as DNA methylation, histone modifications, and noncoding RNAs (ncRNAs) have been shown to regulate genes associated with inflammation, apoptosis, fibrosis, and cardiac repair. These epigenetic alterations contribute to the complex gene-environment interactions that influence clinical outcomes in MI patients. Recent research has identified specific epigenetic changes that can serve as biomarkers for MI risk stratification, offering potential for early diagnosis and personalized therapeutic interventions. Moreover, targeting these epigenetic modifications holds promise as a therapeutic strategy to reduce myocardial damage, enhance cardiac function, and prevent adverse remodeling after MI. This review explores the mechanisms by which epigenetic regulation influences MI pathogenesis and discusses the therapeutic potential of targeting these pathways to improve patient outcomes. By integrating epigenetic therapies into clinical practice, it may be possible to revolutionize the treatment of MI, addressing the disease at its molecular roots and offering more effective, durable interventions.

## Introduction

Cardiovascular diseases (CVD) including myocardial infarction (MI) are among the significant causes of morbidity and mortality in the world [1]. It basically means irreversible death of heart tissue owing to prolonged ischemia, usually caused by an imbalance in the myocardium between supply and demand for oxygen [2]. According to the World Health Organization (WHO), CVDs, among which myocardial infarction is one, were still estimated to cause 17.9 million deaths per year and remain the leading cause of death worldwide [3]. This large global burden points at why effective interventions on the prevention and management of MI are needed [4][5]. Epigenetics, which involves heritable changes in gene expression that do not alter the underlying DNA sequence, has emerged as a crucial regulator of CVDs [6].

The principal epigenetic mechanisms identified in the regulation of gene expression include DNA methylation, histone modifications, and the activity of noncoding RNAs [7][8]. These mechanisms play a critical role in regulating gene transcription, thereby significantly influencing the pathophysiology of cardiovascular diseases [9][10]. In the context of MI, these epigenetic alterations provide a foundational framework for complex gene-environment interactions that contribute to disease development and influence clinical outcomes [11].

Animal studies examining the epigenetic landscape in MI have revealed significant factors that contribute to the exacerbation of the disease [12][13]. However, there is limit clinical trials. This review aims to explore the major epigenetic mechanisms that contribute to the pathogenesis of MI and to identify potential biomarkers that can aid in the early diagnosis and prognosis of MI. Additionally, it presents the therapeutic potential of targeting these epigenetic pathways as a novel approach to treating this life-threatening condition.

## 1. Epigenetic Mechanisms

**Figure-1:**
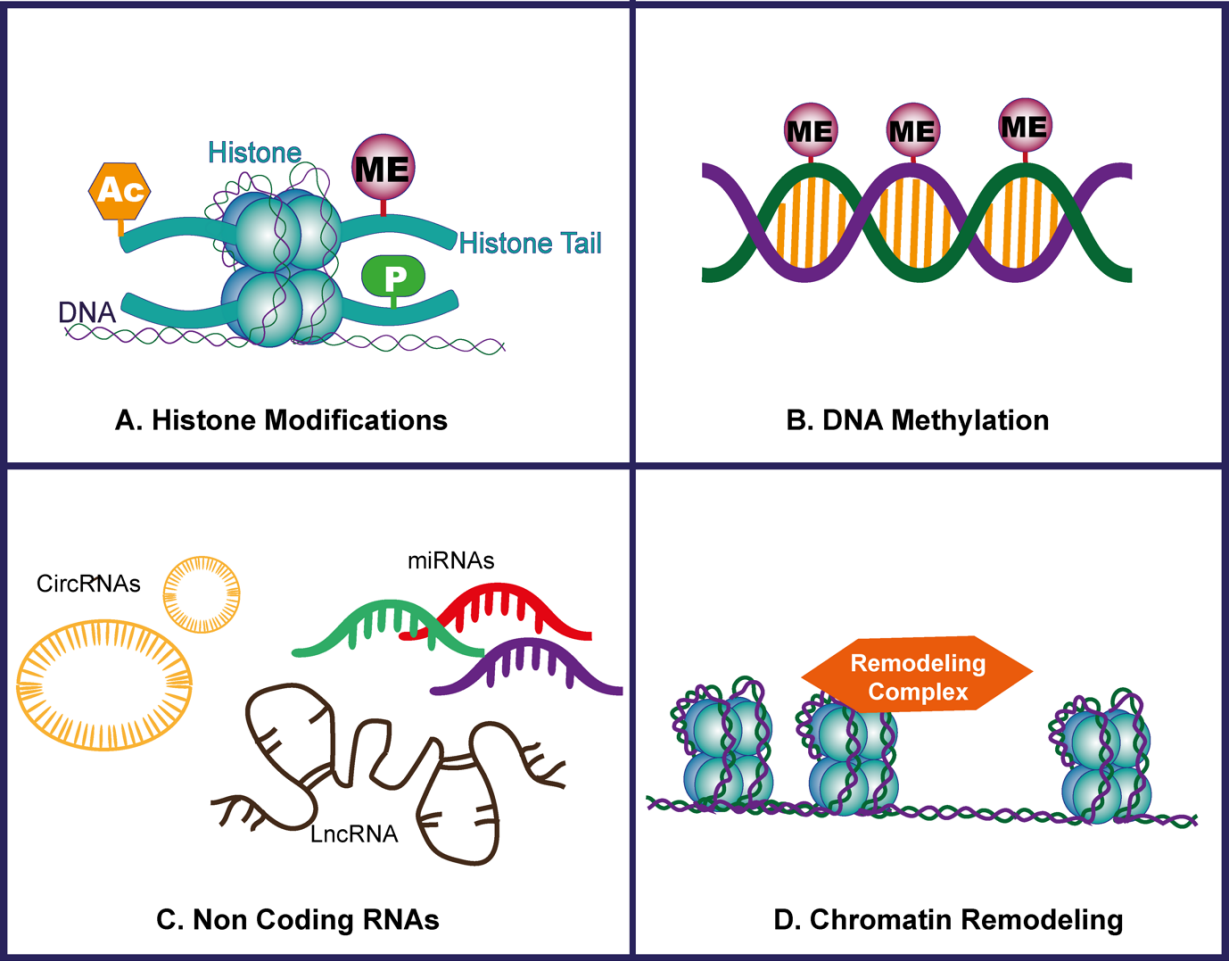
Figure 1. (A) Histone Modifications: This panel illustrates various histone modifications, such as acetylation and methylation, that influence chromatin structure and gene expression by altering the accessibility of DNA to transcription factors. (B) DNA Methylation: This section highlights the process of DNA methylation, where methyl groups are added to the cytosine residues of CpG islands, leading to gene silencing or activation depending on the context and location within the genome. (C) Non-coding RNA: Depicted here are non-coding RNAs, including microRNAs and long non-coding RNAs, which regulate gene expression post-transcriptionally by targeting mRNA for degradation or inhibiting translation, playing a crucial role in cellular processes. (D) Chromatin Remodeling: This panel represents chromatin remodeling complexes that reposition, eject, or restructure nucleosomes, thereby modulating the accessibility of DNA to the transcriptional machinery and influencing gene expression patterns.

Epigenetic mechanisms are considered to influence the pathophysiology of MI through the regulation of gene expression without any alteration in the DNA sequence [7][14].

Figure-1 showed a schematic of common Epigenetic mechanisms. As an example, DNA methylation has been described to alter the response of cardiac ischemic injury through silencing or activation of genes related to inflammation, fibrosis, and apoptosis involved in the process of myocardial infarction.

1.1. DNA Methylation

DNA methylation is an epigenetic process in which a methyl group is added to the 5' position of cytosine residues within CpG dinucleotides in the DNA sequence [15][16]. The event, for the most part, leads to repression of genes by preventing binding of transcription factors to DNA [16]. Alternatively, through recruiting proteins, compactly structures chromatin which makes DNA less accessible for transcription [17]. Unregulated DNA methylation could impact the risk of MI or disease progress [18][19]. Abnormal methylation patterns, such as hypermethylation or hypomethylation, can alter gene expression, potentially contributing to the development of MI by influencing genes involved in inflammation, vascular function, and lipid metabolism [12][18][20].

Hypermethylation typically occurs in gene promoter regions and is associated with the silencing of gene expression. In the context of disease, hypermethylation can suppress the expression of critical genes [21]. In MI, the hypermethylation of certain genes is associated with an increased risk of MI and can exacerbate disease progression [12][19].

Han et al.[12] demonstrated The progression of MI is influenced by the downregulation of energy metabolism genes and the upregulation of genes involved in immune regulation, inflammation, and apoptosis, with hypermethylation of the Tnni3 gene potentially exacerbating the disease. Also, Chen et al. [22] showed there are significant genetic differences in DNA methylation that are linked to disease progression. Moreover, Talens et al. [19] reported Women's risk of MI has been linked to DNA methylation at specific loci influenced by prenatal conditions, implying that epigenetic changes early in life could affect their likelihood of developing MI later on. On the other hand, Hypomethylation generally results in the activation or overexpression of genes, particularly those that are otherwise tightly regulated [12]. While gene activation is necessary for normal physiological processes, aberrant hypomethylation can lead to the pathological overexpression of genes involved in inflammation, immune responses, and fibrosis [23][24]. Tarazón et al. [25] reported genome-wide hypomethylation in the DNA of ischemic cardiomyopathy, alongside dysregulation in the processes involved in the addition, removal, and maintenance of methyl groups. Also, Luttmer et al.[20] demonstrated that DNA hypomethylation is associated with hyperglycemia and low levels of high-density lipoprotein (HDL), both of which are linked to an increased risk of MI [26].

Indeed, these papers suggest that DNA methylation plays a crucial role in regulating gene expression, which impacts myocardial function and the response to injury, thereby increasing the risk and progression of MI [12][22][25]. Targeting these epigenetic modifications indeed offers a very promising therapeutic strategy for mitigating damage from MI and bettering outcomes [17][27].

1.2. Histone Modifications

Histone modifications are epigenetic regulators that perturb chromatin structure in gene-expression-impinging ways. Histones are the protein components of chromatin [28]. Histones can be modified at their tails by several chemical modifications like acetylation, methylation, phosphorylation, ubiquitination, and sumoylation [11]. These modifications would then relax or compact chromatin structure, giving accessibility to specific regions of the genome and making them more or less accessible to other regulatory proteins, including transcription factors [7]. Acetylation of histones, particularly on lysine residues, is typically associated with transcriptional activation [29]. The addition of acetyl groups neutralizes the positive charge on histones, reducing their affinity for DNA and allowing for a more relaxed chromatin structure that is accessible to transcription factors [29][30]. Some studies demonstrated the dysregulation of histone acetylation can lead to aberrant expression of genes involved in endothelial function, inflammatory responses, and hypertrophic signaling, contributing to the pathogenesis of MI and heart failure [31][32]. In contrast, histone deacetylation, mediated by histone deacetylases (HDACs), generally results in transcriptional repression and has been linked to maladaptive cardiac remodeling and fibrosis following MI [33][34][35]. HDACs activity plays a pivotal role in the specification of mesodermal cells into cardiomyoblasts, a process essential for cardiac healing and regeneration [36]. Also, HDACs improve diastolic function and reduce cardiac fibrosis by downregulation miR-133a wich induce pressure overload [37]. Histone methylation, which can either activate or repress transcription depending on the specific amino acid residue and the number of methyl groups added, is also critically involved in cardiovascular health and disease [38]. For example, trimethylation of histone H3 at lysine 27 (H3K27me3) is associated with gene silencing and has been implicated in the suppression of protective genes in cardiac tissues, while H3K4me3, an activating mark, is often found at promoters of genes involved in cardiomyocyte survival and function [39][40]. Aberrant histone methylation patterns have been linked to a range of cardiovascular pathologies, including atherosclerosis, hypertension, and cardiac hypertrophy [37][38][41][42]. Given their central role in gene regulation, histone modifications represent promising therapeutic targets for the treatment and prevention of CVDs [33].

1.3. Non-coding RNAs (microRNAs, lncRNAs)

Non-coding RNAs, in the form of microRNAs (miRNAs) and long non-coding RNAs (lncRNAs), are key post-transcriptional regulators that control the expression of targeted genes without being translated into proteins [27]. These molecules are critical regulators of various physiological processes, including the pathogenesis of MI [43].

1.4. microRNAs

miRNAs, which are short, around 22-nucleotide-long RNA molecules, usually function by binding to complementary sequences in the 3' untranslated regions of target mRNAs, leading to their degradation or translation blockage [44]. miRNAs have emerged as critical regulators of cardiac pathophysiology, influencing processes such as apoptosis, inflammation, fibrosis, and angiogenesis [10]. The dysregulation of specific miRNAs has been implicated in both the acute phase of MI and the subsequent remodeling and repair processes, making them potential biomarkers and therapeutic targets in CVDs [45]. Several miRNAs are notably upregulated or downregulated in response to MI. For instance, Takaya et al. [46] demonstrated miR-1 and miR-133, which are involved in cardiomyocyte differentiation and proliferation, are typically downregulated following MI, leading to impaired cardiac function and increased susceptibility to arrhythmias [47]. Conversely, miR-21 and miR-29 have been found to be upregulated in the post-MI heart, where they contribute to fibrosis and adverse remodeling by targeting genes involved in extracellular matrix production and inflammatory pathways [48][49] The modulation of these miRNAs has shown promise in preclinical studies, where either inhibiting or mimicking their activity can mitigate the pathological effects of MI, highlighting their therapeutic potential [50]. In addition to their roles as regulators of cardiac pathology, miRNAs also hold promise as biomarkers for the diagnosis and prognosis of MI [51]. Circulating levels of certain miRNAs increase significantly in the bloodstream shortly after myocardial injury, reflecting ongoing cardiac damage [52][53]. These miRNAs offer potential as non-invasive biomarkers for early MI detection, risk stratification, and monitoring of therapeutic efficacy [51][54].

1.5. LncRNAs

LncRNAs, which are transcripts longer than 200 nucleotides that do not encode proteins, exert their effects through various mechanisms, including chromatin remodeling, transcriptional regulation, and post-transcriptional modulation [55] LncRNAs play an essential role in modulating the response to ischemic injury, promoting or inhibiting myocardial repair, and affecting the outcome of MI through the control of diverse signaling pathways [56][57][58]. Several investigations showed LncRNA MALAT1 protect cardiomyocytes from ischemic injury by regulating apoptosis and promoting angiogenesis, thereby enhancing cardiac repair [59][60]. Conversely, lncRNAs such as ANRIL have been associated with adverse outcomes in MI, contributing to inflammation and atherosclerotic plaque formation, which exacerbates myocardial damage [61][62]. Moreover, Liu et al. [58] reported that the expression of the human homolog of mouse lncRNA (LncHrt) is reduced in patients with dilated cardiomyopathy. These findings suggest that LncHrt functions as a crucial regulator of cardiac metabolism, playing a vital role in maintaining heart function by modulating key metabolic signaling pathways. Additionally, Du et al. [57] demonstrated that lncRNA (N1LR) acts as a protective factor against MI by regulating the TGF-β/Smads signaling pathway. Similarly, Niu et al. [56] highlighted the critical role of lncRNA (Oip5-as1) in preventing excessive mitochondrial fission during MI. LncRNAs represent a critical and largely untapped resource in the understanding and treatment of myocardial infarction. Their roles as regulators of gene expression and cellular processes position them as valuable biomarkers and therapeutic targets in CVDs [56][57][58].

1.6. Chromatin Remodeling

Chromatin remodeling is a dynamic process that involves changes in chromatin architecture, which subsequently controls the accessibility of DNA to transcription factors and other regulatory proteins [63]. This process plays a crucial role in regulating gene expression, and its dysregulation has been implicated in various pathological conditions [63][64] Chromatin remodeling is increasingly recognized as a critical factor influencing cardiac gene expression, contributing to both heart development and response to ischemic injury. [65][66][67] During ischemia, the heart undergoes significant stress, leading to the activation of various chromatin remodeling complexes. [66][67] These complexes modify histones and alter nucleosome positioning, facilitating or repressing the transcription of genes involved in cardiomyocyte survival, inflammation, fibrosis, and apoptosis [65]. As an example, the SWI/SNF complex has been implicated in the regulation of genes that promote cardiac hypertrophy and fibrosis, processes that are central to the maladaptive remodeling that follows MI [27][68]. Understanding the mechanisms of chromatin remodeling in the heart not only provides insights into the molecular underpinnings of MI but also opens up new avenues for therapeutic intervention aimed at improving recovery and preventing heart failure.

## 2. Epigenetic Biomarkers

Epigenetic biomarkers have emerged as a promising frontier in the diagnosis, prognosis, and management of MI [69]. Unlike genetic mutations, epigenetic modifications do not change the DNA sequence but instead regulate gene expression in a dynamic and reversible manner [45].

2.1. Diagnostic Biomarkers

Epigenetic biomarkers have gained significant attention for their potential in improving the diagnosis of MI [51]. Among these, DNA methylation patterns and specific miRNAs have emerged as promising candidates [45]. Modification of DNA methylation at specific CpG sites in genes such as GNAS and ZNF365 has been associated with MI, offering a potential tool for early diagnosis [70]. Also, circulating miRNAs been identified as sensitive and specific markers of myocardial injury. These miRNAs are released into the bloodstream following cardiomyocyte damage, providing a non-invasive means to detect MI [52][53]. The utility of these epigenetic biomarkers lies in their ability to complement traditional diagnostic approaches, such as cardiac troponin levels and electrocardiograms, by offering additional insights into the molecular events underlying MI [45][51].

2.2. Prognostic Biomarkers

Prognostic biomarkers are essential for predicting the outcomes of patients with MI and guiding therapeutic decisions [54]. DNA methylation signatures and miRNA profiles have shown promise in this area. Qin et al. [71] showed DNA methylation has been linked to adverse cardiac remodeling and poor prognosis following MI. They also identified DNA methylation prognostic genes, such as FKBP5, UBE2E2, and AUTS, which are associated with cellular senescence, myocyte inflammation, and HDL levels, contributing to the adverse outcomes of MI [71]. Similarly, elevated levels of miR-21 and miR-29 in circulation have been associated with increased fibrosis and a higher risk of heart failure [48][49]. Also, Scărlătescu et al. [72] the measurement the level of miR-223-3p, miR-142-3p and miR-146a-5p could be useful as prognostic markers for adverse events of MI. Moreover, Zheng et al. [73] and Chen et al. [74] demonstrated that lncRNAs increased in patients with MI and may serve as potential biomarkers for predicting patient prognosis and cardiac fibrosis. These biomarkers not only provide insights into the likelihood of adverse outcomes but also help stratify patients based on their risk profile, enabling more personalized treatment strategies [54][71]. The identification of reliable prognostic biomarkers could lead to improved long-term management of MI patients, reducing the incidence of complications and enhancing survival rates.

2.3. Methodological Approaches

The identification and validation of epigenetic biomarkers involve a range of advanced technologies and methodologies [75]. DNA methylation analysis typically utilizes techniques such as bisulfite sequencing, which converts unmethylated cytosines to uracil, allowing for the precise mapping of methylation sites [70] . Other approaches, such as methylation-specific PCR and pyrosequencing, are also employed for more targeted analyses [76][77][78] . For miRNA profiling, next-generation sequencing (NGS) and quantitative real-time PCR (qRT-PCR) are commonly used to identify and quantify miRNAs in both tissue samples and circulating fluids [79][80][81]. Additionally, high-throughput methods like microarray analysis facilitate the simultaneous examination of multiple miRNAs, enabling the discovery of novel biomarkers [69][82] .Validation of these biomarkers requires robust statistical analysis and replication in independent cohorts to ensure their reliability and clinical utility [9][45][69]. The integration of bioinformatics tools is also crucial for analyzing large datasets and identifying relevant epigenetic patterns associated with MI.

## 3. Therapeutic Potential of Targeting Epigenetics

3.1. Pharmacological Modulators

The modulation of epigenetic mechanisms through pharmacological agents represents a promising approach to treating MI [83]. Drugs targeting DNA methyltransferases and HDACs are at the forefront of this therapeutic strategy [84]. DNA methyltransferases inhibitors, such as 5-azacytidine, prevent the addition of methyl groups to DNA, thereby reactivating the expression of silenced cardioprotective genes [85]. These inhibitors have shown potential in preclinical models of MI by reducing cardiac fibrosis and improving myocardial function [86]. On the other hand, HDAC inhibitors by preventing the deacetylation of histones, leading to a more relaxed chromatin structure and enhanced expression of genes involved in cell survival, angiogenesis, and anti-inflammatory pathways [87] . Preclinical studies have demonstrated that HDAC inhibitors can reduce infarct size, prevent adverse remodeling, and improve overall cardiac function after MI [88][89]. Therapies targeting non-coding RNAs show significant potential in treating CVDs [90]. Preclinical studies have demonstrated that blocking or mimicking specific ncRNAs can effectively inhibit the progression of atherosclerotic plaques, limit myocardial necrosis, and prevent adverse cardiac remodeling [46][91][92] furthermore, other ncRNAs such Small interfering RNA (siRNA) significantly reduced plasma level of lipoprotein(a) that is useful in management of CVDs [93][94]. However, substantial challenges remain, particularly the unpredictable long-term effects in a diverse human population, making the design of appropriate clinical trials difficult [90]. The ongoing research into these pharmacological modulators highlights their potential to modulate epigenetic landscapes in MI.

3.2. Gene Therapy Approaches

Gene therapy represents a cutting-edge strategy for epigenetic modification in myocardial infarction, with the potential to achieve long-lasting effects on gene expression [95] .In this method, the CRISPR-Cas9 system has emerged as a powerful tool for precise epigenetic editing [96]. By targeting the epigenetic regulators of key genes involved in myocardial injury and repair, CRISPR-Cas9 can be used to activate or silence specific genes to promote cardioprotection [96][97]. Additionally, CRISPR-based approaches can modify histone marks to either activate or repress gene expression in cardiomyocytes, potentially reducing cell death and promoting tissue repair [96]. While still in its early stages, the application of CRISPR-Cas9 for epigenetic editing in MI holds significant promise, offering a targeted and potentially curative approach to mitigating the damage caused by ischemic injury [97].

## 4. Challenges and Opportunities

Despite the exciting potential of targeting epigenetics in MI, several challenges must be addressed to translate these therapies from the laboratory to clinical practice [98][99]. One of the primary challenges is the specificity of epigenetic therapies. Since epigenetic modifications are widespread and occur in many different cell types, ensuring that therapeutic interventions target only the affected cardiac cells without off-target effects is crucial [99]. Another challenge is the delivery of epigenetic drugs or gene therapy vectors to the heart in a safe and efficient manner [100]. Currently, systemic delivery methods may lead to unintended effects in non-cardiac tissues, necessitating the development of more targeted delivery systems. Additionally, the long-term effects of epigenetic modifications are not yet fully understood, raising concerns about potential unforeseen consequences [90][100]. However, these challenges also present opportunities for innovation. Advances in nanotechnology could enable more precise delivery of epigenetic modulators to the heart [15][101]. Furthermore, the ongoing development of more specific and tunable epigenetic editing tools could enhance the safety and efficacy of these therapies. As research progresses, overcoming these challenges will be critical for realizing the full therapeutic potential of epigenetic interventions in MI.

## Conclusion

Epigenetics plays a pivotal role in the pathogenesis, progression, and potential treatment of MI. Through mechanisms such as DNA methylation, histone modifications, and non-coding RNAs, epigenetic alterations significantly influence gene expression, affecting processes like inflammation, fibrosis, and cardiomyocyte survival. These modifications provide a deeper understanding of the molecular underpinnings of MI, offering novel insights into both diagnostic and prognostic biomarkers. Moreover, the therapeutic potential of targeting epigenetic mechanisms, whether through pharmacological agents or gene therapy approaches like CRISPR-Cas9, holds promise for improving MI outcomes. However, translating these epigenetic interventions into clinical practice presents challenges, including specificity, delivery, and long-term safety. Due to these challenges through innovative research and technological advancements will be crucial in harnessing the full therapeutic potential of epigenetics in the treatment of MI, potentially leading to more effective and personalized approaches to cardiovascular care.

## Conflict of Interest

None declared.
